# Efficacy and safety of cannabidiol in a single-center pediatric drug-resistant epilepsy cohort: a retrospective study

**DOI:** 10.3389/fneur.2025.1616480

**Published:** 2025-07-16

**Authors:** Ambra Butera, Giulia Spoto, Graziana Ceraolo, Maria Grella, Ivana Giunta, Maria Ludovica Albertini, Carla Consoli, Caterina Sferro, Maria Spanò, Gabriella Di Rosa, Antonio Gennaro Nicotera

**Affiliations:** ^1^Unit of Child Neurology and Psychiatry, Department of Chemical, Biological, Farmaceutical and Environmental Science, University of Messina, Messina, Italy; ^2^Unit of Child Neurology and Psychiatry, Department of Human Pathology of the Adult and Developmental Age “Gaetano Barresi”, University of Messina, Messina, Italy; ^3^Unit of Child Neurology and Psychiatry, Department of Biomedical Sciences, Dental Sciences and Morphofunctional Imaging, University of Messina, Messina, Italy; ^4^Unit of Child Neurology and Psychiatry, Maternal-Infantile Department, University of Messina, Messina, Italy

**Keywords:** cannabidiol, CB1 receptor, drug-resistant epilepsy, focal cortical development, focal cortical dysplasia

## Abstract

**Background:**

Pharmacoresistance to conventional antiseizure medications has been described in approximately 30% of the pediatric epileptic patients, making pharmacological management particularly challenging for physicians. Currently, cannabidiol (CBD) is approved as an adjunctive therapy in combination with clobazam for Dravet Syndrome (DS), Lennox–Gastaut Syndrome (LGS), and as adjunctive treatment for Tuberous Sclerosis Complex (TSC). Studies on drug-resistant epilepsy (DRE) suggested that CBD antiepileptic properties may benefit a wider range of pharmacoresistant epilepsy syndromes.

**Objective:**

Our observational, retrospective, monocentric study aimed to evaluate the effect and safety of CBD in a real-world pediatric cohort with DRE.

**Methods:**

We recruited 15 pediatric patients (7 females, 8 males; mean age: 12.33 ± 4.37 years) affected by pharmacoresistant epilepsy treated with CBD as adjunctive therapy. Inclusion criteria required a diagnosis of DRE, initiation of CBD treatment before 18 years of age, and at least 6 months period of follow-up after CBD initiation. Clinical, demographic, and instrumental data were retrospectively extracted from the medical records and caregivers’ reports. Based on seizure reduction, patients were stratified into “responders” (>50%), “partial responders” (30–50%), and “non-responders” (<30%) groups.

**Results:**

CBD was used as an add-on therapy in 8/15 patients on-label (for DS, LGS, and TSC) and in 7/15 off-label. The maximum dose of CBD administered was 21 mg/kg/day, with an average dose of 16.5 mg/kg/day. 11/15 patients showed a reduction in seizure frequency: 7 were responders (2/7 seizure-free) and 5 were partial responders. Additionally, 11/15 patients showed improved social and environmental participation, as assessed using the Clinical Global Impression scale. Interestingly, brain magnetic resonance imaging revealed structural abnormalities in 5/15 patients, with 6/15 showing malformations of cortical development (4/6 responders, including 1 seizure-free).

**Conclusion:**

CBD demonstrated a good safety and tolerability profile and appeared to be a promising therapeutic option for the management of DRE. It offers a valuable alternative for seizure control and has a positive impact on social interaction, with overall improvement in the quality of life for patients and their caregivers.

## 1 Introduction

Drug-resistant epilepsy (DRE), as defined by the International League Against Epilepsy (ILAE), is the failure of adequate trials of two tolerated, appropriately chosen and used antiseizure medication (ASM) schedules (whether as monotherapies or in combination) to achieve sustained seizure freedom (1, 2). DRE is observed in approximately 30% of pediatric patients, making pharmacological management particularly challenging for physicians (3).

To date, several new ASMs have been introduced, offering improved efficacy and safety profiles for epilepsy treatment, and have been trialed especially in drug-resistant and catastrophic conditions (4–6). The research on the endocannabinoid system has suggested a potential role in epileptogenesis, indicating that exogenously produced cannabinoids may have antiseizure effects in treating DRE (3, 7). There is growing interest in the antiepileptic properties of cannabis-derived products, especially on the two main components of cannabis, Δ9-tetrahydrocannabinol (THC) and cannabidiol (CBD) (7). While THC is a psychoactive agent, and its effect on seizures remains controversial, basing on its effect of exacerbating seizure activity, CBD is a non-psychoactive phytocannabinoid that has shown efficacy in several placebo-controlled trials (8–14). Nevertheless, the exact mechanisms underlying its antiseizure properties in human and animal models are still not fully understood (8). Numerous molecular targets have been proposed for CBD, and it can exert different pharmacological effects by interacting with different receptors (15). CBD effects are mainly mediated through G protein coupled receptors, known as cannabinoid type 1 (CB1) and type 2 (CB2), which are highly expressed in the central nervous system, particularly in hippocampus (7, 16). However, the modulation of ion channels such as gamma-aminobutyric acid (GABA)_A_ receptor and voltage-gated channels (i.e., sodium Na_V_ and calcium Ca_V_) has been implicated in the anticonvulsant and antiepileptic effects of CBD, as well as its interaction with the G protein-coupled receptor 55 (GPR55) receptor and the transient receptor potential cation channel subfamily V (TRPV1) calcium channel (15).

CBD is currently approved in numerous countries, including Italy, as adjunctive antiseizure therapy in combination with clobazam for Dravet Syndrome (DS) and Lennox–Gastaut Syndrome (LGS), and as adjunctive therapy for Tuberous Sclerosis Complex (TSC) (17, 18). DS is a developmental and epileptic encephalopathy (DEE) caused by mutation in SCN1A gene. It is typically resistant to conventional ASMs, with onset in the first year of life (usually around 6 months) and is associated with negative prognostic features such as cognitive, motor, often behavioral impairment, and early mortality (19). However, randomized controlled trials on patients with DS demonstrated a significant reduction in seizure frequency (>50%) with add-on CBD therapy compared to placebo (9, 11).

LGS is a severe epileptic encephalopathy, characterized by multiple seizure types (mainly generalized), a typical electroencephalogram (EEG) interictal pattern with diffuse slow spike-and-wave complexes (1.5–2.5 Hz) and generalized paroxysmal fast activity, and cognitive impairment that typically progresses to intellectual disability (12). In patients with LGS, CBD in add-on to conventional ASMs demonstrated a significant and sustained efficacy in reducing seizure frequency, particularly drop-attack episodes (10, 12).

TSC is an autosomal dominant genetic disorder caused by mutations in the *TSC1* or *TSC2* genes, which regulate cell growth and proliferation. Pathogenic variants in these genes result in a spectrum of features, including benign hamartomas in multiple organ systems, often accompanied by infantile DRE and subsequent neurological issues, such as cognitive and neuropsychiatric disorders (14). The use of CBD in patients with TSC significantly decreased generalized and focal seizure frequency, also leading to dose reductions of most concomitant ASMs compared to baseline (13, 14).

Although CBD is widely used in pediatric epilepsy treatment, pharmacokinetic data in infants and children remain limited (8). Recent real-world studies have started to expand the potential therapeutic use of highly purified CBD beyond the currently approved indications (3, 20). A large multicenter observational study on 266 patients with genetically confirmed monogenic epilepsies showed that 47.5% of patients achieved a ≥ 50% reduction in seizure frequency, with similar effectiveness observed both in approved conditions (DS, LGS, TSC) and in off-label use for other genetic epilepsies (21). These findings support the role of CBD as a promising treatment option also in other DEEs, including rare and highly refractory forms. This observational, retrospective, monocentric study aims to evaluate the effect and assess the safety/tolerability of CBD in a real-world pediatric cohort affected by DRE.

## 2 Materials and methods

We conducted a retrospective observational study on our monocentric pediatric cohort of 15 subjects affected by pharmacoresistant epilepsy, who were treated with CBD and presented to the Unit of Child Neurology and Psychiatry at the “G. Martino” Polyclinic Hospital in Messina over the past 4 years. The cohort consisted of 7 females and 8 males, all of whom had received CBD as adjunctive therapy.

Inclusion criteria required patients to be under 18 years of age at the initiation of CBD therapy, have a confirmed diagnosis of DRE, defined according to ILAE criteria as the failure of adequate trials of two tolerated and appropriately chosen ASMs (as monotherapy or in combination) to achieve sustained seizure freedom (1). Patients had to be on CBD treatment for a minimum period of 6 months.

CBD was administered as an oral solution, starting at an initial dose of 5 mg/kg/day, with titration based on clinical response and tolerability. As this was a retrospective study, the clinical response was assessed by individual clinicians during follow-up visits basing on parents’ reports of the seizures frequency and was considered collectively in our analysis.

Clinical, demographic, and instrumental data were retrospectively extracted from the medical records and caregivers’ reports. Information collected included neurocognitive development, epilepsy characteristics (age at onset, seizure semiology, ASMs used), and response to CBD treatment (tolerability and side effects). Baseline seizure frequency was determined based on parent’s report at the last follow-up visit prior CBD initiation. Seizures were classified according to their onset and type, based on the predominant manifestation, in accordance with the ILAE classification (22). Brain magnetic resonance imaging (MRI) results and relevant data from any genetic testing were also analyzed.

The assessment of socio-environmental participation was evaluated using the Clinical Global Impression (CGI) scale, a standardized tool used to measure the overall change in the clinical status of patients.

Based on seizure reduction, patients were stratified into 3 response subgroups: “responders” (>50%), “partial responders” (30–50%), and “non-responders” (<30%).

In patients with polymorphic seizures, response classification was based on the overall reduction in seizure frequency. Additionally, seizure reduction was assessed separately according to seizure onset and semiology.

Descriptive statistics were reported as frequencies (sample size and percentages) and medians (minimum and maximum) for categorical and continuous variables. To evaluate whether the presence of malformations of cortical development was significantly associated with an increased rate of clinical response to CBD treatment, a one-tailed Fisher’s exact test was conducted.

## 3 Results

This study describes a pediatric cohort of 15 patients (mean age: 12.33 ± 4.37 years; m:f = 8:7) affected by DRE treated with CBD (Table 1). All patients had a seizure onset at a mean age of 17.07 months (range: 0–96 months) and had previously undergone pharmacological treatment with other ASMs up to therapeutic doses, with partial or limited benefit. We included patients that received a stable regimen of ASMs, tailored to their clinical needs, combined with CBD as an adjunctive therapy. The treatment approach was optimized for each patient, incorporating a combination of CBD and other ASMs, aiming to achieve better seizure control and improve quality of life. CBD was administered in combination with a median of three ASMs, including clobazam in 8/15 of the cases.

**Table 1:** Clinical features of our cohort.

Patient (sex, age)	Diagnosis	CL	Age at epilepsy onset	ST at epilepsy onset	Actual ST	Brain MRI	Actual therapy	Max CBD dosage mg/kg/day	RR	Side Effects	Social and environmental response	Genetic testing
**1** **(m, 8 yo)**	**LGS**	**ID**	**3 yo**	**G** **T, A**	**G** **T, A**	**Bilateral subcortical band heterotopia** **(> left frontal)**	**CBZ, CLB,** **CBD 17 mg/kg/day**	**21**	**>50%**	**Yes** **(irritability)**	**Positive**	**Not informative WES**
2 (m, 20 yo)	LGS	ID	8 yo	G T, Ab	G A, T, Es	Not Informative	VPA, LTG, CLB, VNS, CBD 20 mg/kg/day	20	30–50%	Yes (irritability)	None	SHANK3 Pathogenic Variant
3 (f, 17 yo)	LGS	ID	5 mo	F, G T, Es	F, G T, A, Es	Malformative (rotation abnormalities in the occipital-temporo-mesial site and CC thinning)	VPA, LCS, CLB, CBD 18 mg/kg/day	18	30–50%	Yes (irritability)	Positive	Ongoing WES
4 (m, 9 yo)	LGS	ID	1 yo	G T	F, G T, Es	Lissencephaly	VPA, ETH, CBD 9 mg/kg/day	10	30–50%	No	Positive	LIS1 Pathogenic Variant
5 (m, 11 yo)	LGS	ID	2 mo	G (also SE) T	G T, A, Ab, Es	Porencephaly (hypoxic–ischemic lesions)	FFA, VPA, LCS	21	<30%	No	None	Ongoing WES
**6** **(m, 16 yo)**	**DS**	**ID**	**5 mo**	**G** **feb/afeb T and Es**	**Seizure- Free**	**Not Informative**	**STP, CLB, VPA,** **CBD 7 mg/kg/day**	**10**	**Seizure-free**	**Yes,** **(GI symptoms)**	**None**	**SCN1A Pathogenic Variant**
7 (m, 16 yo)	DS	ID	6 mo	G, F feb/afeb T (also SE)	G T, Es	Hippocampal morphological alterations	VPA, CLB, STP, CBD 18 mg/kg/day	18	<30%	No	Positive	SCN1A del
**8** **(f, 9 yo)**	**TSC**	**ID**	**8 mo**	**G, F** **Es, Au**	**F** **Au**	**TSC typical MRI pattern (cortical tubers, subependymal nodules, WM abnormalities) and CC thinning.**	**CBZ, PER,** **CBD 15 mg/kg/day**	**15**	**> 50%**	**No**	**None**	**TSC2 Pathogenic Variant**
**9** **(f, 10 yo)**	**DRE**	**ID**	**3 mo**	**G** **T, Es**	**Seizure- Free**	**Frontal polymicrogyria and CC agenesis**	**LCS, CLB,** **CBD 19 mg/kg/day**	**19**	**Seizure-free**	**No**	**Positive**	**Not informative WES**
**10** **(f, 15 yo)**	**DRE**	**ID**	**4 yo**	**G, F** **T, A, Au**	**F** **Au**	**Bilateral parietal FCD**	**CBZ, VPA, CLB,** **CBD 15 mg/kg/day**	**15**	**>50%**	**No**	**Positive**	**Ongoing WES**
**11** **(f, 14 yo)**	**DRE**	**ID**	**0 yo**	**G** **Tc, Es** **(also SE)**	**G (last seizures)** **Tc**	**Malformative** **(CC thinning, brainstem atrophy, periventricular WM atrophy, ON hypoplasia)**	**LEV, PB, CBZ,** **CBD 20 mg/kg/day**	**20**	**>50%**	**No**	**Positive**	**Atypical WBS** **YWHAG del**
**12** **(m, 12 yo)**	**DRE**	**ID**	**2 yo**	**G** **T**	**G** **T, Ab**	**Hippocampal morphological alterations**	**VPA, ETH,** **CBD 7 mg/kg/day**	**10**	**>50%**	**No**	**Positive**	**Not Informative WES**
13 (m, 8 yo)	DRE	ID	1 mo	G T	G T, Es	Malformative (WM atrophy, CC thinning, cerebellar hypoplasia with atypical hemispheres gyri)	VPA, CLB, LCS, CBD 17 mg/kg/day	17	30–50%	Yes (irritability)	Positive	Not Informative WES
14 (f, 16 yo)	DRE	ID	9 mo	G T, Es	G T, A, Ab	Not informative	VPA, FFA, ETH, CBD 14 mg/kg/day	14	30–50%	No	Positive	SETBP1 Pathogenic Variant
15 (f, 4 yo)	DRE	DD	1 mo	G A, Es	G A, Es	Not Informative	LCS, CZP, CBD 21 mg/kg/day	21	<30%	Yes (irritability)	Positive	CDKL5 Pathogenic Variant

In bold are the patients with a response >50%.

<, lesser than; >, greater than; A, atonic seizures; Ab, atypical absence seizures; Au, autonomic seizures; CBD, cannabidiol; CBZ, carbamazepine; CC, corpus callosum; CL, cognitive level; CLB, clobazam; CZP, clonazepam; DD, developmental delay; del, deletion; DRE, drug-resistant epilepsy; DS, Dravet Syndrome; Es, epileptic spasms; ETH, ethosuximide; f, female; F, Focal; feb/afeb, febrile/afebrile; FCD, focal cortical dysplasia; FFA, fenfluramine; G, Generalized; ID, intellectual disability; LCS, lacosamide; LEV, levetiracetam; LGS, Lennox–Gastaut Syndrome; m, male; mo, months; MRI, magnetic resonance imaging; ON, optic nerves; PB, phenobarbital; PER, perampanel; RR, response rate; SE, status epilepticus; ST, seizure type; STP, stiripentol; T, tonic seizures; Tc, tonic–clonic seizures; TSC, tuberous sclerosis complex; VPA, valproic acid; WBS, Williams-Beuren Syndrome; WES, whole exome sequencing; WM, white matter; yo, years old.

The cohort included 5/15 patients diagnosed with LGS, 2/15 with DS, 1/15 with TSC, and 7/15 with other forms of DRE. Data regarding the clinical history, including age and seizure type at epilepsy onset, are summarized in Table 1.

**Figure 1:**
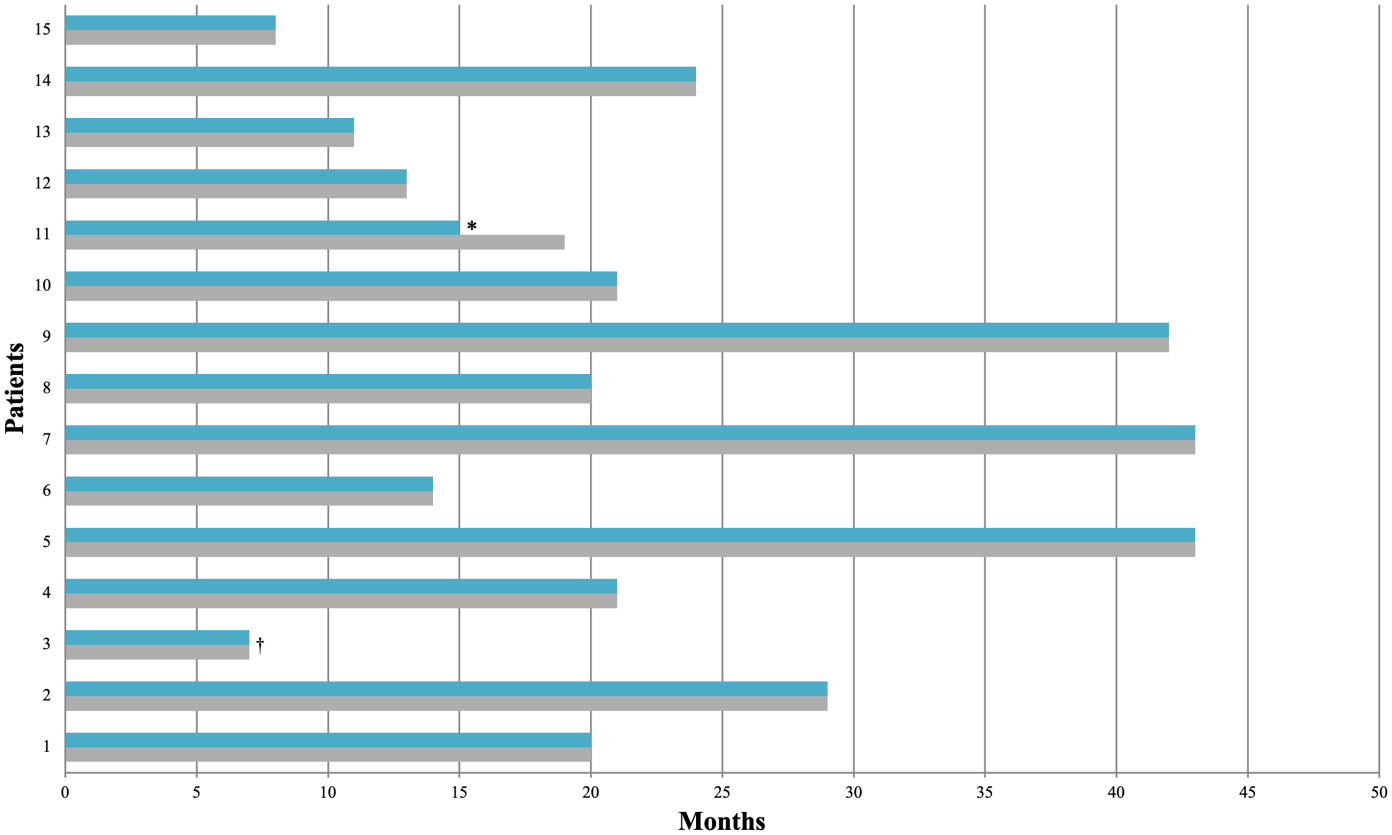
CBD treatment duration. †, the patient died due to the underlying condition; *, drop out. In light blue is the duration of CBD treatment, while in gray is the duration of follow-up.

CBD was administered as an add-on therapy in 8/15 patients on-label (for LGS, DS, and TSC) and in 7/15 off-label, with appropriate titration. The maximum dose of CBD was 21 mg/kg/day, with an average dose of 16.5 mg/kg/day. The mean duration of CBD treatment was 22 months (range 7–43 months) (Figure 1).

**Figure 2:**
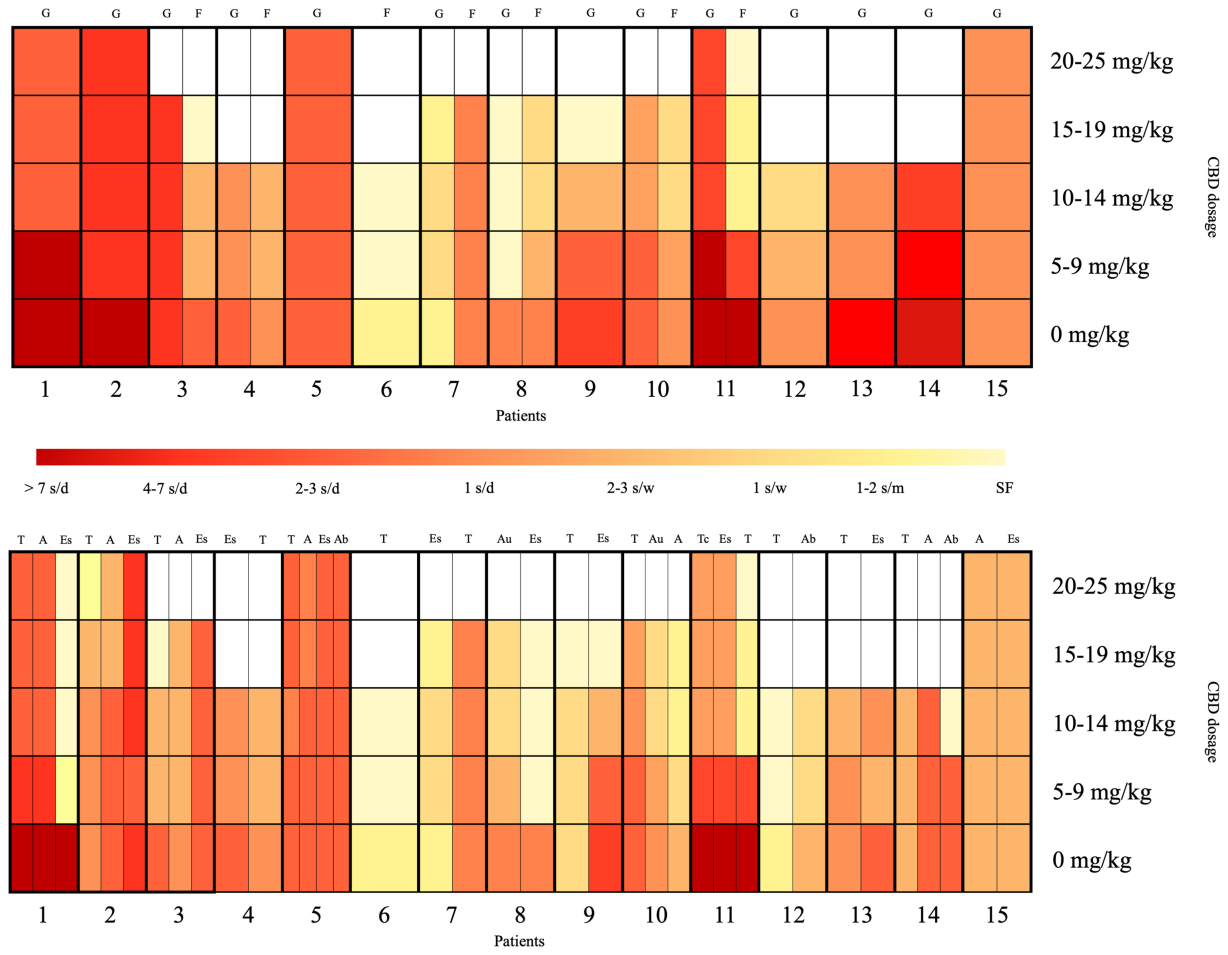
Visual summary of the clinical response to CBD, stratified according to seizure types and CBD dosage. The x-axis represents the 15 patients, and the y-axis shows the CBD dosage per day. The red-to-yellow gradient reflects seizure frequency, ranging from dark red (>7 seizures/day) to light yellow (seizure-free). The upper section of the heatmap displays the seizure response classified by onset type (focal vs. generalized), while the lower section shows the response categorized by specific seizure types. A, atonic seizures; Ab, atypical absence seizures; Au, autonomic seizures; d, day; Es, epileptic spasms; F, focal seizures; G, generalized seizures; m, month; s, seizures; SF, seizure-free; T, tonic seizures; Tc, tonic–clonic seizures; w, week.

Figure 2 depicts a visual summary of the clinical response to CBD, stratifying patients according to seizure types. Generalized seizures were reported in 14/15 patients and showed an overall good response: 6/14 patients had a response >50%, 4/14 had a partial response (30–50%), and 4/14 had a response <30%. A good response was also observed in focal seizures (described in 7/15 cases), with 5/7 patients showing a response >50% (case 6 being seizure-free), 1/7 showing a 30–50% response, and 1/7 with a response <30%.

Among seizure types, tonic seizures were most frequently reported (13/15) and showed a response >50% in 8/13 patients, including complete remission in 5/8 cases; two case (2/13) showed a 30–50% response, while the remaining 3/13 had a response classified as <30%. Epileptic spasms were reported in 11/15 patients, with 4/11 showing a response >50% (including complete remission in 3 cases); two cases (2/11) showed a 50% response, classified as partial response, while the remaining 5/11 had a response <30%. Atonic seizures were present in 7/15 patients and showed an overall good response: 2/7 had a response >50%, 3/7 had a 30–50% response, and only 2/7 had a response <30%. Atypical absences were reported in 3/15 patients, with a seizure reduction >50% in 2/3 cases (case 14 in remission), while the third patient (1/3) showed a response <30%. Autonomic seizures were present in 2/15 patients, both (2/2) showing a response >50%. Tonic–clonic seizures were reported in only 1/15 patient and showed a response >50%.

At the last follow-up, 13/15 patients continued to experience seizures: 2/13 had focal seizures, 9/13 had generalized seizures, 2/13 had both generalized and focal seizures.

Overall, 11/15 patients showed a reduction in seizure episodes: 7/15 patients were responders (1/7 LGS, 1/7 DS, 1/7 TSC, 4/7 DRE), of whom 2/7 were seizure-free. Among them, one patient diagnosed with DS has been seizure-free for 2 years, while the other, affected by DRE, has been seizure-free for approximately 10 months. 5/15 patients were partial responders (2/5 DRE, 3/5 LGS). The remaining 3/15 patients did not show a tangible benefit in terms of seizure recurrence; however, 2/3 of these patients experienced improvements in socio-environmental participation.

In total, 11/15 patients demonstrated enhanced socio-environmental participation (assessed using the CGI scale). Only one patient (1/15) showed no improvement in either seizure control or social engagement, resulting in a drop out after 15 months. One patient, classified as a responder (see Table 1), died during active CBD treatment due to complications related to the underlying syndrome.

**Figure 3:**
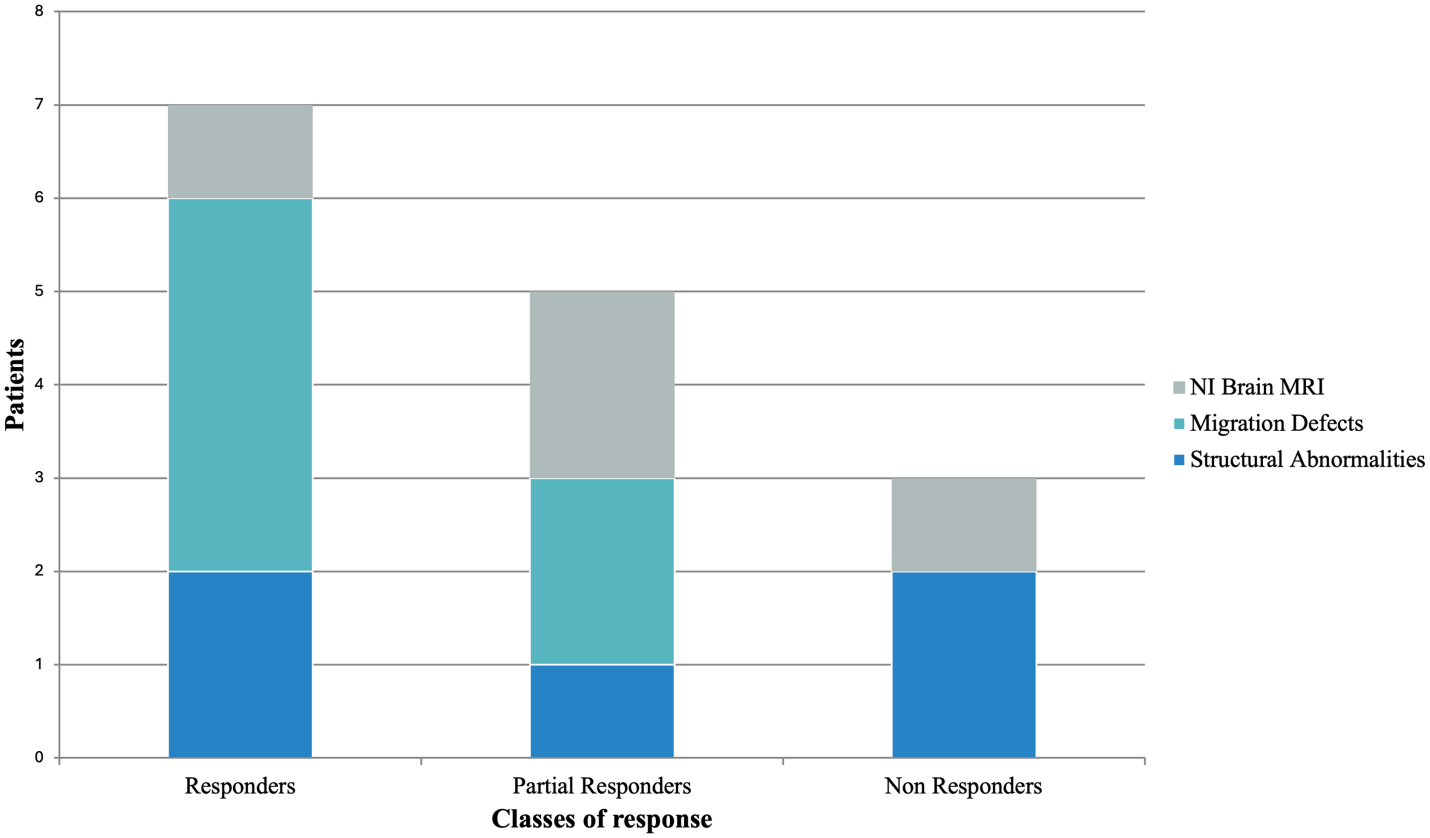
Brain MRI findings according to CBD response stratification. MRI, magnetic resonance imaging; NI, not informative.

Neuroimaging studies were not informative in 4/15 cases. In the remaining 11/15, MRI scans revealed structural abnormalities (5/15) and evidence of malformations of cortical development (6/15); among these latter, 4/6 were classified as responders (including 1/4 seizure-free) and 2/6 as partial responders (Figure 3). A comparison of clinical response between patients with and without malformations of cortical development yielded a *p*-value of 0.1846.

Regarding side effects, 6/15 patients reported mild and transient manifestations: 5 experienced irritability/hyperactivity and 1 reported gastrointestinal symptoms (diarrhea).

All subjects (15/15) exhibited cognitive impairment on standardized evaluations: 1/15 with developmental delay and 14/15 with moderate to severe intellectual disability.

Genetic testing was performed in all patients, revealing causative variants in 8/15: 1/15 Atypical Williams-Beuren Syndrome due to a large 7q11.23 deletion (including the *YWHAG* gene) detected by array-Comparative Genomic Hybridization (aCGH); 1/15 *SETBP1* identified by targeted trio-based Next-Generation Sequencing (NGS) panel; 2/15 *SCN1A* (patient 6 carried a pathogenic variant revealed by a targeted trio-based NGS panel, while patient 7 presented a deletion detected by aCGH); 1/15 *SHANK3* identified by trio-based Whole Exome Sequencing (WES); 1/15 *CDKL5* revealed by trio-based WES; 1/15 *LIS1* deletion detected by aCGH; 1/15 *TSC2* pathogenic variant already present in other members of the family and identified through Sanger sequencing. Moreover, 3/15 had ongoing trio-based WES at the time of data collection, whereas in 4/15 the trio-based WES was not informative.

## 4 Discussion

CBD is currently approved as adjunctive antiseizure therapy for DS and LGS in combination with clobazam, and as adjunctive therapy for TSC (12, 14, 19). However, many authors have investigated its potential role in other forms of DRE, where its use is off label. Indeed, literature data suggested that CBD antiseizure properties may be extended to a much broader spectrum of DREs (3, 20). CBD has demonstrated benefits in other epilepsy syndromes, both as long-term ASM and as treatment of acute manifestations (i.e., refractory status epilepticus) (23, 24). Over the years, clinical practice has provided clear evidence that CBD serves as an effective alternative for controlling seizures in patients with these pharmacoresistant epileptic conditions (18).

Our pediatric cohort of pharmacoresistant epilepsies included 7/15 (46.7%) DRE which were treated off label. Overall, 73.3% of cases (responders and partial responders) showed a reduction in seizure frequency with CBD treatment; in particular, 46.7% of patients achieved a significant reduction (>50%) in seizures (7/15 responders), with two cases displaying seizure freedom. Notably, none of the patients reported worsening of seizure frequency. These data were consistent with those reported in the literature. Particularly, real-world studies highlighted a significantly reduction in seizure frequency, with rates ranging from 36.9 to 68.8% of the patients investigated (23, 25), and were further confirmed by recent multicenter evidence supporting the efficacy of CBD in both approved and off-label genetic epilepsies (21). In line with the literature, drop seizures—defined as seizures characterized by an increase or decrease in muscle tone that leads or could lead to a fall, including tonic, atonic, and tonic–clonic seizures—were the types that showed the best response in terms of seizure reduction, with 5 out of 8 patients (62.5%) achieving complete remission from tonic seizures (9, 10, 26, 27).

In our sample, 8 patients were treated with a combination of clobazam and CBD, which demonstrated a good effectiveness in reducing seizure frequency. Specifically, 7/8 patients —comprising 3 with LGS, 1 with DS, and 3 with DRE — were identified as responders or partial responders. It has been proposed that the potential bidirectional drug interaction between CBD and clobazam may influence the overall effect and safety of CBD as an antiepileptic therapy, increasing the exposure to active metabolites of both drugs (28). Therefore, concomitant therapy with CBD and clobazam has been associated with a greater reduction in seizure frequency but also a higher incidence of side effects (18).

Although the mechanisms underlying CBD effects in humans are still not fully understood, evidence suggested that its action is multimodal, involving both cannabinoid and non-cannabinoid pathways, which may account for its strong anti-inflammatory and neuroprotective properties (15, 29). About 60 different neurological molecular targets of CBD have been identified, including enzymes, ion channels, and both ionotropic and metabotropic receptors (15, 29). Particularly, the endogenous cannabinoid system (including CB1 and CB2 receptors), the GPR55 receptor, and the TRPV1 calcium channel have been implicated in the CBD role as ASM (30).

Noteworthy, malformations of cortical development were observed in 6 of our patients: patient 1 had bilateral subcortical band heterotopia; patient 3 displayed a malformative pattern, including abnormalities in cortical gyration of the occipital-temporo-mesial regions; patient 4 showed lissencephaly; patient 8 had cortical tubers, consistent with the typical TSC neuroimaging pattern; patient 9 presented with frontal polymicrogyria in the context of Aicardi syndrome; and patient 10 exhibited bilateral parietal focal cortical dysplasia (FCD). It is crucial to point out that all these patients showed a good response to CBD, with 66.6% (4 out of 6 cases) experiencing a > 50% reduction in seizures, representing 57.1% of all the “best” responders (4 out of 7 cases – patients 1, 8, 9, and 10); the remaining 2 patients (33.3%) showed a seizure reduction between 30 and 50%, accounting for 40% of all partial responders (2 out of 5 patients – patients 3 and 4) (Figure 3). Malformations of cortical development are a heterogeneous group of brain malformations caused by disorders in neurogenesis, neuronal migration, post-migration neuronal development, and cortical organization. Over 75% of the patients with malformations of cortical development develop seizures over the course of their life, and in 40% of the cases present with pharmacoresistant epilepsies (31). Genetic factors play an important role in their etiology. Particularly, pathogenic variants of genes encoding regulators of the mammalian target of rapamycin (mTOR) cascade have been implicated in the occurrence of epilepsies, malformations of cortical development, and neurodevelopmental disorders (32). The involvement of mTOR pathway has been proved in FCD, in polymicrogyria, and in the TSC manifestations (31, 32). In fact, since they share histopathological features with FCD type II, cortical and subcortical tubers in TSC have been reclassified as FCD22 (33). FCD type II display an increase in CB1 receptor expression levels, especially in neurons with overactive mTOR Complex 1 (mTORC1) signaling (34). In addition, experiments conducted on Xenopus oocytes micro-transplanted with surgical and post-mortem tissue samples (including FCD type IIb) from DRE patients demonstrated that CBD has a modulatory effect on GABAergic currents. The authors suggested that this higher effect could be linked to specific cellular subtypes or different configurations of the GABA_A_ receptors (35). All this evidence may suggest that the receptor profile of patients with malformations of cortical development, characterized by increased CB1 receptor expression levels and a different configuration of the GABA_A_ receptors, favors the antiseizure effect of CBD, explaining the good response to treatment observed in this group within our cohort. To further explore this observation, we performed a Fisher’s exact test to evaluate whether patients with malformations of cortical development were more likely to respond to CBD. The analysis showed a trend toward significance, though it did not reach statistical thresholds, likely due to the limited sample size. These preliminary findings should be interpreted with caution, but they support the hypothesis of a differential treatment response in this subgroup and may serve as a foundation for future studies in larger, more homogeneous populations. Moreover, one of our patients, who did not have an evident malformation of cortical development, presented a *de novo* very large deletion including the *YWHAG* gene, which encodes for the tyrosine 3-monooxygenase/tryptophan 5-monooxygenase activation protein gamma. *YWHAG* is involved in the mitogen-activated protein kinases (MAPK) pathway through the RAF-1 bond and the mTORC1 signaling pathway, and its haploinsufficiency can lead to an altered expression of CB1 receptors in neurons (36). This patient showed a good response (>50% reduction of seizure) to CBD treatment, further enforcing the idea that genetic variants affecting the mTORC1 pathway may present a favorable profile of CB1 receptors that positively influence the clinical response to CBD treatment, similarly to malformations of cortical development such as FCD and TSC.

In addition to these molecular interactions, CBD has been shown to induce notable changes in brain connectivity. Specifically, studies have highlighted modifications in resting-state functional connectivity, particularly in inter-regional connections between the vermis, amygdala, hippocampus, and frontal cortex. Moreover, CBD appears to influence attentional processes by modulating the functional connectivity between the superior frontal gyrus and the insula/middle frontal gyrus (37, 38). Our study demonstrated significant benefits of CBD treatment, over seizure reduction, and improved social participation. Interestingly, the observed improvements in social functioning also contribute to better behavioral profiles. This is particularly interesting given that ASMs, especially in polytherapy, may show an adverse effect on the cognitive profile (39, 40). However, directly evaluating behavioral outcomes in our patient cohort proved challenging. Nonetheless, previous studies have already suggested a positive role of CBD in addressing neuropsychiatric symptoms (41, 42).

In our series, CBD treatment was generally well tolerated. The most common adverse event was transient irritability/hyperactivity (33%), which was dose-dependent or occurred during the titration phase; gastrointestinal disturbances were recorded in only one case (6.7%). Our results are in line with the literature, as side effects were reported in 10–47% of patients and encompassed somnolence, decreased appetite, diarrhea, transaminase elevations, fatigue, rash, sleep disorders, and infections (23, 29). In our cohort, only one patient has discontinued CBD treatment due to a lack of clinical benefit (6.7%). Cases of treatment discontinuation due to lack of effect have already been reported in other studies in literature; real-world studies have documented discontinuation in 37% of treated patients (43).

This study has several limitations that should be considered when interpreting the findings. The retrospective design may introduce biases related to data collection and the reliance on medical records and caregiver reports, potentially affecting the accuracy and consistency of the findings. The small sample size, which reduces statistical power, limits the further stratification by clinical variables (i.e., genetic etiology, epilepsy type) and affects the generalizability of our findings to the broader pediatric DRE population. Although improvements in seizure frequency and socio-environmental participation were documented, the assessment relied on subjective tools such as the CGI scale. In retrospective studies, this subjectivity—combined with potentially incomplete data—may increase the risk of bias. Another limitation lies in the heterogeneity of the cohort, including differences in genetic profiles and baseline characteristics, which complicates the interpretation of treatment response variability. Furthermore, the retrospective design also precluded monitoring of CBD metabolite serum levels (i.e., 7-OH-CBD, 7-COOH-CBD, 6-OH-CBD), limiting correlations between pharmacokinetics and clinical outcomes (44). Moreover, technical details of the genetic analyses, such as sequencing depth, were not available in the medical records, further limiting the ability to fully characterize the molecular findings. Given the role of several genes in epileptic encephalopathies (45), further research is also needed to investigate the correlation between genetic variants and treatment outcomes in order to implement more effective and personalized treatment approaches. Finally, the single-center nature of this study limits their applicability to broader populations with varying access to healthcare and treatment protocols. Future multicenter, prospective studies with larger sample sizes and standardized methodologies are necessary to validate these results.

## 5 Conclusion

Although these results are based on a limited sample size, they support the implementation of CBD as an add-on treatment in pharmacoresistant epileptic syndromes. Future research should focus on exploring the use of CBD in DREs beyond the currently approved indications, such as LGS, DS, and TSC. Additionally, the existing literature on the effect of CBD in DREs involving malformations of cortical development is currently limited, and further studies should be conducted to confirm this correlation. Expanding the application of CBD to other forms of DRE, including those with malformations of cortical development or complex genetic etiologies, could uncover additional therapeutic benefits and broaden its clinical utility.

Future frontiers of research should focus on achieving the deep understanding of CBD-related effects on seizures, as well as its action on environmental participation and overall neuropsychiatric comorbidities that could positively affect also on behavioral and social skills outcomes.

In conclusion, CBD may represent a promising therapeutic option capable of enhancing the clinical management of DRE. Indeed, it offers a valuable alternative for seizure treatment and positively impacts social interaction and quality of life for the patients and their caregivers.

## Data Availability

The original contributions presented in the study are included in the article/supplementary material, further inquiries can be directed to the corresponding author.

## Glossary

ASM: antiseizure medication
CB1: cannabinoid type 1
CB2: cannabinoid type 2
CBD: cannabidiol
CBZ: carbamazepine
CC: corpus callosum
CGI: clinical global impression
CL: cognitive level
CLB: clobazam
CZP: clonazepam
DD: developmental delay
DEE: Developmental and Epileptic Encephalopathies
del: deletion
DRE: drug-resistant epilepsy
DS: Dravet Syndrome
EEG: electroencephalogram
ESp: Epileptic Spasms
ETH: ethosuximide
F: female
feb/afeb: febrile/afebrile
FCD: focal cortical dysplasia
FFA: fenfluramine
G: Generalized
GABA: gamma-aminobutyric acid
GPR55: G protein-coupled receptor 55
ID: intellectual disability
LCS: lacosamide
LEV: levetiracetam
LGS: Lennox–Gastaut Syndrome
m: male
MAPK: mitogen-activated protein kinases
mo: months
MRI: magnetic resonance imaging
mTOR: mammalian target of rapamycin
mTORC1: mammalian target of rapamycin Complex 1
ON: optic nerves
PB: phenobarbital
PER: perampanel
RAF-1: RAF1 protooncogene, serine/threonine kinase
RR: response rate
SE: status epilepticus
ST: seizure type
STP: stiripentol
TSC: tuberous sclerosis complex
THC: tetrahydrocannabinol
TRPV1: transient receptor potential cation channel subfamily V
VPA: valproic acid
WBS: Williams-Beuren Syndrome
WES: whole exome sequencing
WM: white matter
YO: years old
